# Matrix metalloproteinase 9 (MMP-9) is indispensable for long term potentiation in the central and basal but not in the lateral nucleus of the amygdala

**DOI:** 10.3389/fncel.2015.00073

**Published:** 2015-03-11

**Authors:** Tomasz Gorkiewicz, Marcin Balcerzyk, Leszek Kaczmarek, Ewelina Knapska

**Affiliations:** ^1^Department of Neurophysiology, Nencki Institute of Experimental Biology, Polish Academy of SciencesWarszawa, Poland; ^2^Department of Biophysics, Warsaw University of Life SciencesWarszawa, Poland; ^3^Department of Molecular and Cellular Neurobiology, Nencki Institute of Experimental Biology, Polish Academy of SciencesWarszawa, Poland; ^4^Unidad Ciclotron, Centro Nacional de Aceleradores (Universidad de Sevilla – CSIC – Junta de Andalucia)Sevilla, Spain

**Keywords:** LTP, MMP-9, amygdala, synaptic plasticity, learning

## Abstract

It has been shown that matrix metalloproteinase 9 (MMP-9) is required for synaptic plasticity, learning and memory. In particular, MMP-9 involvement in long-term potentiation (LTP, the model of synaptic plasticity) in the hippocampus and prefrontal cortex has previously been demonstrated. Recent data suggest the role of MMP-9 in amygdala-dependent learning and memory. Nothing is known, however, about its physiological correlates in the specific pathways in the amygdala. In the present study we show that LTP in the basal and central but not lateral amygdala (LA) is affected by MMP-9 knock-out. The MMP-9 dependency of LTP was confirmed in brain slices treated with a specific MMP-9 inhibitor. The results suggest that MMP-9 plays different roles in synaptic plasticity in different nuclei of the amygdala.

## Introduction

Matrix metalloproteinase-9 (MMP-9) is a member of a matrix metalloproteinase family of zinc-dependent extracellular and membrane bound endopeptidases that cleave components of the extracellular matrix. Its activity is tightly controlled by an endogenous inhibitor, tissue inhibitor of matrix metalloproteinases 1 (TIMP-1, Dziembowska and Wlodarczyk, 2012). MMP-9 has been shown to be involved in synaptic plasticity, as well as in learning and memory involving the hippocampal formation. In particular, it has been observed that MMP-9 knock-out mice display a deficit in late phase of long term potentiation (L-LTP), but not in its early phase (E-LTP) in the hippocampal CA3 to CA1 pathway (Nagy et al., 2006). Similarly, chemical inhibition of MMP-9 causes destabilization of LTP in the mossy fibers-CA3 pathway (Wojtowicz and Mozrzymas, 2010). Moreover, deficits in spatial learning and contextual fear conditioning were observed in MMP-9 knock-out mice (Nagy et al., 2006). It has also been demonstrated that overexpression of TIMP-1 *in vivo*, as well as specific chemical inhibition of MMP-9 in acute slice preparation block late phase of LTP in the subiculum to medial prefrontal cortex pathway (Okulski et al., 2007). On the other hand, very little is known about the role of MMP-9 in neuronal plasticity in other brain structures, including the amygdala. Nagy et al. (2006), as well as Brown et al. (2009) reported that interfering with MMP-9 activity did not affect amygdala-dependent fear conditioning to an acoustic cue. Recently, we have shown that appetitively, but not aversively motivated discrimination learning depends on MMP-9 activity within the central amygdala (Knapska et al., 2013).

Little is also known about the molecular heterogeneity corresponding to the function of the amygdalar nuclei. It has, however, been well established that this brain structure is complex, with over a dozen subdivisions distinguished by anatomical, as well as functional features (see: Sah et al., 2003; Knapska et al., 2007). Such functional heterogeneity suggests that different molecular mechanisms may underlie processing of information in different nuclei of the amygdala. For instance, studies on expression of gene activity markers in response to different kinds of behavioral training have revealed patterns of activation specific to the learning task (see Knapska et al., 2007).

To address the question about molecular heterogeneity of the amygdalar nuclei at the level of synaptic plasticity we investigated the role of MMP-9 in LTP measured at three different amygdalar pathways: from the external capsule (EC) to the lateral amygdala (LA), from the LA to the basal amygdala (BA) and from the BA to the medial section of the central amygdala (CeAm). We used coronal brain slices from mice lacking *mmp-9* gene (MMP-9 KO). The slices were subjected to a tetanic stimulation protocol that produces both early (E-) LTP and, a subsequently emerging, protein synthesis-dependent late (L-) LTP. Additionally, we induced LTP in the pathways from the EC to LA and from the BA to CeAm in rat brain slices treated with S24994, a chemical inhibitor of MMP-9.

## Materials and Methods

**Figure 1:**
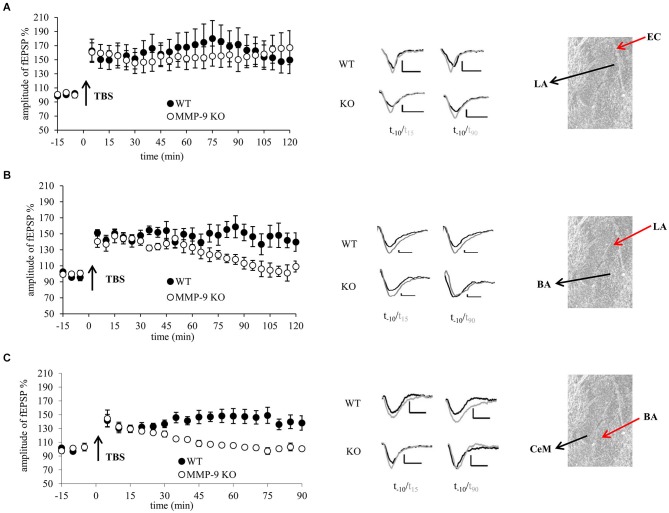
**Genetic inhibition of MMP-9 results in destabilization of LTP in the central and basal but not in the lateral amygdala. (A)** fEPSP in the EC–LA amygdala pathway was similar in slices from mice lacking functional MMP-9 gene (MMP-9 KO, open circles *n* = 6) and control animals (WT, filled circles, *n* = 5). **(B)** fEPSP evoked in the LA-BA pathway in slices from MMP-9 KO mice (open circles, *n* = 7) within first 70 min had the same magnitude as LTP in slices from control animals (WT, filled circles, *n* = 7); however afterwards it went down to the baseline level. **(C)** fEPSP induced in the BA-CeAm pathway in slices from MMP-9 KO mice (open circles, *n* = 7) had the same amplitude as LTP evoked in control slices (filled circles, *n* = 7) within first 30 min after induction. Then, LTP in MMP-9 KO slices gradually decreased to the baseline level. Left panels show graphs with time course of maximal EPSP amplitudes normalized to baseline. Black arrows mark the time of application of TBS stimulation. Error bars represent SEM. Middle panels show exemplary traces of fEPSP recorded 10 min before (black) and 15 and 90 min after (gray) induction of LTP. Scale bars = 0.2 mV and 5 ms. Right panels present photographs of mouse amygdala (Nissl staining) with positions of stimulating (red arrow) and recording (black arrow) electrodes.

MMP-9 homozygous knock-out mice on a C57BL/6 background were obtained from Dr. Z. Werb (University of California, San Francisco). These mice were bred with C57BL/6NtacF wild-type mice for at least two generations and then maintained and bred continuously with each other as heterozygotes for >10 generations. The MMP-9 KO and MMP-9 WT mice used in this study were always littermates. The experiments were performed on male 2- to 4 month-old mice. For experiments with MMP-9 inhibitor 2- to 3-month-old male Wistar rats were used. All of the animals were group-housed and maintained on a 12 h/12 h light/dark cycle with water and food provided *ad libitum*. The animals were treated in accordance with the ethical standards of European (directive no. 86/609/EEC) and Polish regulations resulting from this directive. All of the experimental procedures were approved by the Local Ethics Committee. Animals were anesthetized with isoflurane and decapitated. The brains were quickly removed and placed in cold artificial cerebrospinal fluid (aCSF; 117 mM NaCl, 4.7 mM KCl, 2.5 mM NaHCO_3_, 1.2 mM NaH_2_PO_4_, 2.5 mM CaCl_2_, 1.2 mM MgSO_4_ and 1 mM glucose), bubbled with carbogen (95% O_2_ and 5% CO_2_). Both hemispheres were cut into 400 μm coronal slices with a vibratome. The slices were then transferred to a recording interface chamber and perfused with carbogenated aCSF at 33°C for at least 1 h before the LTP experiments started. Field excitatory postsynaptic potentials (fEPSP) were recorded using glass electrodes (1–3 MΩ resistance). Electrodes positions are shown in Figure 1. Test pulses at 0.033 Hz, 0.1 ms, were delivered by a bipolar metal electrode (FHC). The intensity of a test stimulus was adjusted to obtain fEPSP with amplitude that amounted to a half of the maximal response. After at least 15 min of stable baseline recording, a theta burst protocol (TBS) was used to evoke LTP. Three trains of stimuli were applied every 5 min. One train was composed of five sequences of pulses separated by 1 s. Each sequence consisted of five bursts of stimuli at 5 Hz. The bursts consisted of eight pulses at 100 Hz. After the end of the theta burst protocol, test pulses were subsequently applied for at least 90 min. Recordings were amplified and digitized, and amplitudes were analyzed online and off-line (CED, Cambridge, UK). The same protocol was used in the experiments with S24994 (Hanessian et al., 2001; Jourquin et al., 2003), a specific MMP-9 inhibitor. After 15 min of baseline recordings S24994 was delivered (100 nM), and, 15 min later, TBS protocol was used to induce LTP. S24994 was present in ACSF throughout all remaining recording time. ANOVA with repeated measures was used for statistical analysis of responses averaged in 5 min intervals; *p* < 0.05 was considered significant.

## Results

In the first experiment we examined whether MMP-9 is important for LTP in the LA. We induced LTP by stimulation of the EC, in brain slices from MMP-9 KO and MMP-9 WT mice (Figure 1A). We observed no statistically significant difference between LTP recorded in slices from MMP-9 KO and MMP-9 WT mice (155.1 ± 15.7% of baseline vs. 161.6 ± 18% of baseline).

Next we investigated LTP in the pathway from the LA to BA. TBS stimulation protocol evoked LTP that was stable within first 70 min in slices from both MMP-9 KO (137.2 ± 7.2% of baseline) and MMP-9 WT controls (147 ± 7.4% of baseline). However, thereafter LTP in MMP-9 KO declined to the baseline level, whereas it remained stable in MMP-9 WT mice (repeated measures ANOVA: *F*_(1,12)_ = 5.95, *p* = 0.04 for comparison between two groups for the last 50 min of recording, Figure 1B).

Finally, we investigated impact of MMP-9 knock out on LTP in the CeAm. LTP evoked in slices from MMP-9 KO was stable only within first 30 min (129.7 ± 5.9% of baseline), then it decayed to the baseline level within 15 min. LTP in MMP-9 WT group was stable throughout entire recording period (141 ± 81% of baseline; repeated measures ANOVA: *F*_(1,12)_ = 14.73, *p* = 0.004 for the last 60 min of recording, Figure 1C).

We also investigated basal synaptic transmission in MMP-9 KO and WT mice in all three pathways. There were no differences in I-O relationship between MMP-9 KO and WT mice neither in the EC–LA nor in the LA to the BA and BA to the CeAm amygdala pathways (data not shown).

**Figure 2:**
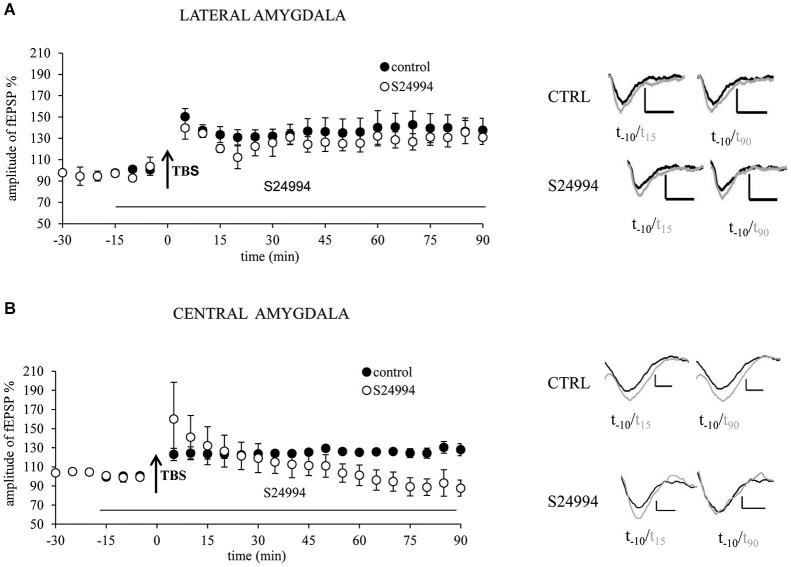
**Chemical inhibition of MMP-9 with highly specific inhibitor S24994 results in destabilization of LTP in the central but not in the lateral amygdala. (A)** MMP-9 inhibitor S24994 (100 nM) administrated 15 min before LTP induction did not affect fEPSP in the EC–LA amygdala pathway (open circles, *n* = 4) in comparison with control, untreated slices (filled circles, *n* = 5). **(B)** MMP-9 inhibitor S24994 (100 nM) administrated 15 min before LTP induction destabilized fEPSP in the BA-CeAm pathway 30 min after its induction (open circles, *n* = 4), whereas fEPSP evoked in control slices was unaffected (filled circles, *n* = 5). Black arrows mark the time of application of TBS stimulation. Error bars represent SEM. Beneath the graphs there are exemplary traces of fEPSP 10 min before (black) and 15 and 90 min after (gray) induction of LTP. Scale bars = 0.2 mV and 5 ms.

To confirm MMP-9 dependency of LTP, we investigated the brain slices treated with S24994, a specific MMP-9 inhibitor. We studied two amygdalar pathways: from the EC to LA and from the BA to CeA. In the LA, LTP in slices treated with S24994 did not significantly differ from LTP obtained in control, untreated slices (128 ± 8.1% of baseline vs. 137.3 ± 10.5% of baseline, Figure 2A). In the CeAm, LTP induced in the presence of MMP-9 inhibitor was very similar to LTP recorded in the control slices within first 30 min (133.4 ± 21% of baseline and 123.4 ± 4.7% of baseline, respectively). However, LTP under MMP-9 inhibition returned to the baseline level within 50 min, whereas, in control slices, LTP remained elevated for at least 90 min (repeated measures ANOVA : *F*_(1,7)_ = 6.824, *p* = 0.039 for the last 60 min of recording, Figure 2B).

### Results Summary

In summary, in the present study we show that LTP in the lateral to basal (LA-BA) and basal to the medial division of the central amygdala (BA-CeAm) pathways in MMP-9 KO mice are disrupted in its late phase, whereas LTP in the cortico-LA pathway remains intact. The results obtained in MMP-9 knock-outs were confirmed in slices treated with specific MMP-9 inhibitor.

## Discussion

LTP in the LA has been proposed as a putative cellular mechanism for fear learning (LeDoux, 2000; Maren, 2005; Whitlock et al., 2006). Specifically, the cortico-LA pathway carrying information from the auditory cortex has been implicated in encoding of acoustically cued fear memory (LeDoux, 1995; Schroeder and Shinnick-Gallagher, 2005). Our electrophysiological data presented here, showing that genetic and chemical inhibition of MMP-9 does not affect long-term plasticity in the LA, are consistent with the observation that mice lacking MMP-9 have no deficits in cued fear conditioning (Nagy et al., 2006). On the other hand, little is known about LTP in the CeA, which is downstream from the sites of associativity in the LA and BA and is most often considered as a primary output nucleus for information processing in the amygdala (Davis, 1986; LeDoux, 2000). Here, we show that MMP-9 is important for stabilization but not for induction of LTP in the BA-CeAm pathway. This result is consistent with our behavioral data on MMP-9 KO mice and mice with blocked MMP-9 activity in the CeA, in which we showed impairments in appetitively but not in aversively motivated discrimination learning (Knapska et al., 2013). Moreover, we observed impaired formation of late phase of LTP in the LA-BA pathway. It has been shown that the BA is involved in learning of different behaviors such as fear conditioning and extinction, and appetitively and aversively motivated instrumental learning (Everitt et al., 2003; Maren, 2005, 2011). The role of MMP-9 in the BA in learning and memory needs further studies.

Although MMP-9 is present and active in all amygdalar nuclei, including the LA, BA and CeAm (Knapska et al., 2013), the present results suggest different role of MMP-9 in various parts of the amygdala. The possible explanation of this phenomenon may be based on the fact that LTP at the BLA-CeAm synapses studied herein has been reported to be independent of GABA inhibition (Fu and Shinnick-Gallagher, 2005) whereas LTP at the EC-LA pathway strictly depends on GABA-ergic modulation (Ehrlich et al., 2009). To date, MMP-9 was found to be present in a subset of dendritic spines bearing asymmetric (i.e., glutamatergic) synapses and was not detected on the synapses that are symmetric and expressing GABAa receptors (Wilczynski et al., 2008; Gawlak et al., 2009). We have also demonstrated a similar synaptic localization of MMP-9 activity in the CeA (Knapska et al., 2013). This may suggest that MMP-9 plays a role in plasticity of glutamatergic rather than inhibitory synapses. In line with this reasoning, MMP-9 was shown to be able to modify kinetics of NMDA receptors (Gorkiewicz et al., 2010), which are located post-synaptically and crucial for post-synaptically induced form of LTP (Lynch, 2004). Finally, it is known that neurons in the BLA send axons that form glutamatergic synapses onto neurons within the CeAm (Pape and Pare, 2010).

### Discussion Summary

In summary, we show that LTP in the basal and central but not in the LA is affected by MMP-9 deficiency. These results suggest functional and molecular diversity between the amygdalar nuclei. There have been some evidence supporting this idea (Savonenko et al., 1999; Knapska et al., 2006, 2007), but in the present study we have shown for the first time that one protein plays different roles in synaptic plasticity in different nuclei of the amygdala.

## Conflict of Interest Statement

The authors declare that the research was conducted in the absence of any commercial or financial relationships that could be construed as a potential conflict of interest.
